# Near-Infrared Imaging in Small Animal Surgical Oncology: Current Applications and Future Directions

**DOI:** 10.3390/ani16101563

**Published:** 2026-05-21

**Authors:** Maureen A. Griffin

**Affiliations:** Department of Clinical Sciences, Colorado State University College of Veterinary Medicine and Biomedical Sciences, 300 W Drake Rd, Fort Collins, CO 80523, USA; maureen.griffin@colostate.edu

**Keywords:** near-infrared imaging, fluorescence, indocyanine green, sentinel lymph node, fluorophore

## Abstract

The use of near-infrared (NIR) fluorescence imaging has become increasingly widespread in the intraoperative management of human cancer patients, with diverse applications that enhance the visualization of tumors, metastatic lesions, draining lymph nodes, and critical anatomy. In recent years, the adoption of NIR imaging in small animal surgical oncology has grown, with reported applications including nonselective and selective tumor imaging as well as sentinel lymph node (SLN) mapping for staging and surgical guidance. Despite these advances, clinical use in veterinary patients remains in its early stages, and further work is needed to define indications, optimize protocols, and determine its impact on outcomes. This review summarizes the current applications of NIR imaging in companion animal oncologic surgery, including techniques for dye administration, imaging agents, and reported clinical uses across tumor types. Key limitations are discussed, including limited tissue penetration, lack of tumor specificity with commonly used agents such as indocyanine green, variability in protocols, and limited data in certain species. Emerging technologies, including targeted fluorescent agents and advanced imaging approaches, are also highlighted. Overall, NIR imaging represents a promising adjunct in small animal surgical oncology, though further study and standardization are needed to support broader clinical integration.

## Simple Summary

Cancer surgery in dogs and cats aims to remove tumors completely while preserving as much normal tissue as possible. However, it can be difficult to clearly identify tumor edges or detect small areas of spread during surgery. Near-infrared imaging uses a special dye and camera system to make certain tissues glow, helping surgeons see tumors and draining lymph nodes in real time during surgery. This review summarizes how this technology is currently being used in veterinary cancer surgery. Studies show it can help locate tumors and guide the removal of important lymph nodes for staging. However, the most commonly used dye is not specific to cancer and may also highlight non-cancerous tissues, which can limit accuracy. In addition, the methods are not yet standardized, and there is limited information in some species, especially cats. Newer, more targeted imaging approaches may improve accuracy in the future. Overall, near-infrared imaging shows promise for improving cancer surgery in veterinary patients, but continued research is needed to fully understand its benefits and optimize its use.

## 1. Introduction

Neoplasia is a leading cause of death in humans and companion animals, and surgical excision of neoplastic lesions remains the mainstay therapeutic modality for many tumors [1,2,3]. The early and accurate intraoperative detection of neoplastic tissues is imperative for improvement in patient outcomes [3]. Common challenges encountered in surgical oncology procedures include the accurate localization of tumors, delineation of tumor margins, and identification of metastatic lesions and lymph nodes. Accurate differentiation between tumor and normal tissue is needed to achieve complete excision without overtreatment and/or damage to important surrounding tissues that would result in excessive morbidity. The appropriate identification of metastatic lesions and lymph nodes for sampling or excision is important in many tumor types to determine prognosis, guide adjuvant therapies, and potentially impact outcomes. Furthermore, given the many reported patient-centered and potential oncologic benefits of minimally invasive surgery (MIS) compared to open surgery, the use of MIS in cancer patients has increased substantially [4,5,6,7]. However, intraoperative detection of neoplastic tissue may be more challenging in these cases due to the lack of tactile feedback [3].

There have been substantial advances in preoperative oncologic imaging modalities. Intraoperatively, the incorporation of adjunctive methods beyond visual inspection and palpation to enhance the identification and localization of neoplastic disease for resection is becoming increasingly important in order to reduce the risk of residual disease and to accurately stage patients without excessive morbidity. A 2018 database study evaluating the ten most common solid organ cancers in people in the United States demonstrated minimal improvement in positive surgical margin rates over a 14-year period (1998–2012) for most cancers, with an increase observed in cancers such as bladder and colorectal tumors [8]. Positive histologic margins may influence recommendations for adjuvant therapy and negatively impact prognosis [8]. In addition to considerations related to primary tumor resection and margin status, studies in several human tumor types (such as non-small cell lung cancer) have shown that, despite modern preoperative staging modalities, a substantial proportion of patients are upstaged due to occult nodal disease identified intraoperatively when thorough lymph node evaluation is performed [9,10]. These findings can significantly alter postoperative treatment recommendations and prognostic assessment. This underscores the importance of routine intraoperative nodal staging for appropriate tumor types. Near-infrared (NIR) imaging has emerged as a valuable intraoperative tool that assists surgeons in localizing primary tumors, accurately delineating margins for complete resection, and identifying metastatic or occult synchronous lesions for improved staging [3].

With NIR fluorescence imaging, a fluorescent agent (fluorophore) is administered systemically or locally. Specialized imaging systems with a light source are used to emit photons that are absorbed by the fluorophore, which then enters an excited state and emits photons with a longer wavelength as it returns to a ground state; the emitted photons are captured by a sensitive charge-coupled device camera and displayed in real time [3]. NIR light (700–900 nm) is used to minimize autofluorescence and enhance the contrast between fluorescent signals in neoplastic and non-neoplastic tissue [3]. While tissue autofluorescence in the ultraviolet to visible light spectrum (350–650 nm) has been explored for tumor visualization, with NIR fluorescence imaging, background tissue autofluorescence is reduced due to the low photon absorption by endogenous biomolecules, thereby resulting in an improved signal-to-background ratio (SBR) and a brighter image on the screen [2]. In addition, the use of NIR light allows for deeper tissue penetration (generally at least 1 cm in depth) compared to visible light (with an approximately 2 mm penetration depth) without concurrently interfering with the surgical field evident in visible light, as NIR light is invisible to the human eye [2,3]. In most NIR imaging systems that are currently used, the NIR light source, visible light, and NIR camera are combined into a single system that projects an NIR fluorescence image onto a screen integrated with visible light images of the field for enhanced intraoperative guidance [3]. These imaging systems have been adapted by many laparoscopy/thoracoscopy and robotic surgery platforms for ease of use in MIS procedures, in which NIR imaging may play a particularly important role in augmenting the intraoperative assessment [3,11].

Two of the most common approaches to NIR imaging in oncologic procedures involve either the systemic or local administration of fluorescent agents, each with distinct objectives. In this review, these techniques are discussed in the context of their respective roles in NIR imaging, along with the specific agents utilized in small animal oncologic applications. The current literature and evidence base for these approaches in companion animal cancer patients are summarized, followed by a discussion of the existing limitations and future directions for NIR imaging in small animal surgical oncology.

## 2. Materials and Methods

A literature search was conducted to identify publications related to applications of NIR imaging in small animal surgical oncology. The databases NCBI-PubMed and Google Scholar were searched without date restrictions, with additional focused searches limited to the most recent five years (2022 through 2026). Search terms included combinations of the following: [(near-infrared imaging) AND (dog OR cat) AND (cancer OR neoplasia); (indocyanine green) AND (dog OR cat) AND (cancer OR neoplasia); (near-infrared imaging) AND (dog OR cat) AND (sentinel lymph node); and (targeted) AND (near-infrared imaging) AND (dog OR cat)]. To be eligible for review, the complete manuscript had to be available in English.

This manuscript represents a narrative review of the literature, and relevant articles were selected and synthesized based on clinical relevance and organized into sections according to NIR imaging agent type and application.

## 3. NIR Applications in Small Animal Surgical Oncology

### 3.1. Systemic Administration of NIR Fluorescent Agents for Primary Tumor and Metastasis Detection

#### 3.1.1. Nonselective NIR Agents: Second Window Imaging with Indocyanine Green

With systemic administration of fluorescent agents, the goal is to utilize intraoperative NIR imaging to identify primary tumors, their margins, and any potential metastatic or satellite lesions [3]. The imaging agent used can be non-targeted, targeted (i.e., with specific binding to a receptor or protein), or activatable via an enzymatic reaction or specific pH [1,12]. The most commonly used, FDA-approved, non-targeted NIR fluorescent imaging agent is indocyanine green (ICG). This organic dye is metabolized through the biliary system and has a very small risk of adverse reactions, with a reported incidence of less than 0.1% in people [13]. When administered intravenously (IV) preoperatively, particularly with delayed imaging protocols (i.e., approximately 24 h preoperatively), ICG accumulates within a variety of solid tumors via the enhanced permeation and retention (EPR) effect. Through this mechanism, ICG passively accumulates in tumor tissues due to increased angiogenesis, defective tumoral endothelial cells, and widened vascular fenestrations; once within the tumor microenvironment, ICG is retained locally as a result of impaired lymphatic drainage and its physicochemical properties, including molecular size, shape, charge, and polarity [1,13]. This approach is commonly referred to as second window indocyanine green (SWIG) imaging, whereby uptake and retention of ICG are leveraged to facilitate intraoperative NIR visualization of primary tumors and potential metastatic lesions [14,15]. The EPR effect may be considered both an advantage of ICG use, in that it can be applicable to a variety of tumor types and disease settings, and also a disadvantage of ICG use, in that ICG is not selective for neoplastic tissues. For instance, inflamed tissue can also appear fluorescent when ICG is administered IV due to similar blood and lymphatic vessels in neoplasia and inflammation with a hyperpermeable status of both [2,16]. In addition, due to the lack of selectivity for neoplastic tissues, poorly vascularized tumors may be difficult to detect with NIR when ICG is used in an SWIG application due to a low tumor-to-background signal [2]. The current literature on SWIG imaging via preoperative IV administration of ICG for delineation of tumors and potential metastatic disease in small animals is summarized below and in Table 1.

SWIG imaging has been reported in a translational study on canine and human soft tissue sarcoma [17,18]. In a preliminary study that included 3 dogs, preoperative IV ICG administration resulted in enhanced NIR fluorescence of the tumor compared to the surrounding tissues in all cases, with negative resection margins in all dogs based on both NIR imaging and histopathology [18]. In a subsequent study on dogs with soft tissue sarcoma, preoperative IV ICG administration resulted in strong NIR fluorescence of 14/15 tumors (following intraoperative dissection for tumor exposure as needed) with a mean SBR of 16 ± 5.1; the dog without strong tumor fluorescence had a myxosarcoma that was comprised largely of myxomatous matrix rather than solid tumor tissue [17]. In this study, NIR imaging of the surgical site following tumor excision was characterized as negative for residual disease if the SBR was <5 and positive if the SBR was >10, and the NIR imaging margins were determined to be discordant from the histologic margins in 2/15 cases (negative in 1 dog, positive in 1 dog) [17]. Also of importance, NIR fluorescence was identified 3–6 mm beyond the tumor edge, which was suspected to be due to diffusion of fluorescent light and was not associated with neoplastic extension on histologic assessment or ICG leakage on immunofluorescence microscopy [17].

In one study on dogs with a variety of superficial solid tumors (including mammary tumors, mast cell tumors, and melanoma), SWIG imaging resulted in a median SBR of 1.4 with differential NIR fluorescence of the tumor and surrounding tissue in 4/9 cases (2 mast cell tumors, mammary adenoma, and mammary extraskeletal osteosarcoma) [19]. Imaging of the surgical bed after tumor excision revealed 9 additional fluorescent lesions, of which only 4 contained tumor tissue on histopathology [19]. Ex vivo NIR-guided dissection was performed in 6 cases with resulting moderate sensitivity (72%) and specificity (80%) in demarcating neoplastic vs. non-neoplastic tissue [19]. Given the small sample size and variety of tumor types, conclusions were unable to be drawn for NIR findings relative to specific tumor type in this study.

In another study on SWIG imaging for canine mammary tumor excision, 28/41 (68.3%) tumors were fluorescent with a mean SBR of 1.5 ± 0.2 [20]. Sensitivity of NIR fluorescence was 80% for all malignant tumors and 93.3% for malignant tumors >2 cm [20]. Specificity of NIR fluorescence was 55% for malignant tumors <2 cm and 30% for tumors >2 cm, and tumors >2 cm were more likely to be fluorescent but not more likely to be malignant compared to smaller tumors [20]. In 5 dogs, there was residual fluorescence in the wound bed following resection, but only 1/5 lesions was identified to contain residual neoplasia on histologic assessment [20]. Of 7 inguinal lymph nodes resected, 4 fluoresced but only 2/4 contained metastatic disease [20].

Two studies to date have evaluated the use of SWIG imaging in dogs with pulmonary tumors [16,21]. In one report on 8 dogs with primary pulmonary tumors, the mean tumor SBR was 8.8 and neoplastic lesions were identified via NIR fluorescence in all cases; importantly, in 3 cases, NIR imaging was not able to distinguish tumor tissue from peritumoral inflammatory tissue [16]. In a larger and more recent study on 40 dogs with 44 pulmonary nodules that received IV ICG preoperatively, NIR fluorescence was observed in all nodules, but the sensitivity and specificity of NIR fluorescence for histologic completeness of resection was only 67.7% and 60%, respectively [21]. When 7 lymph nodes were assessed for NIR fluorescence relative to nodal metastasis, the sensitivity was 100% and specificity was 75% [21]. Investigation of IV administered ICG for intraoperative detection of pulmonary metastatic disease in dogs is underway [22].

Two studies have reported the use of IV ICG with intraoperative NIR imaging to detect hepatic tumors in dogs [23,24]. Importantly, in both of these studies, lower doses of ICG were administered as compared to traditional SWIG applications, due to the potential for strong background NIR fluorescence of liver tissues at higher ICG doses (which could result in a reduced SBR) [25,26]. In a historical study, 12 hepatic masses/nodules (including benign and malignant) were resected from 6 dogs following IV ICG administration [23]. Fluorescence was detected in 4/6 malignant lesions and 1/6 benign lesion, such that the sensitivity, positive predictive value, and specificity of NIR fluorescence for malignancy were 71.4%, 80.0%, and 83.3%, respectively [23]. In a more recent and larger study, preoperative IV ICG was administered in 104 dogs with 122 liver masses/nodules [24]. Of all lesions, 106/122 had increased, 7/122 had the same, and 9/122 had decreased NIR fluorescence relative to normal liver tissue, respectively, and the fluorescence intensity and pattern were not significantly associated with the histologic diagnosis [24]. When NIR fluorescence was evaluated relative to histologic margin assessment in 47 dogs, the sensitivity was 100% and specificity was 77.3% [24].

A single case report has described SWIG imaging in a dog with intestinal adenocarcinoma [27]. In that dog, the tumor exhibited reduced NIR fluorescence as compared to surrounding intestine [27]. No information was provided regarding fluorescence of lymph nodes prior to intraoperative sentinel lymph node (SLN) mapping with ICG, which was also performed [27].

**Table 1:** Small animal surgical oncology publications evaluating SWIG imaging using IV ICG administration with delayed intraoperative NIR imaging.

Manuscript	Species and Number of Animals	Tumor Type	IV ICG Dose	Interval Between ICG Administration and Surgery
Madajewski et al., Clin Cancer Res 2012 [18]	3 dogs	Soft tissue sarcoma	7.5 mg/kg	24 h
Holt et al., J Biomed Opt 2015 [17]	15 dogs	Soft tissue sarcoma	3 mg/kg	24 h
Favril et al., Vet Rec 2020 [19]	9 dogs	Mammary tumor (5: adenocarcinoma in 3, adenoma in 1, extraskeletal osteosarcoma in 1), mast cell tumor (3), melanoma (1)	5 mg/kg	24 h
Newton et al., PLOS One 2020 [20]	16 dogs	Mammary tumors (41 total: 20 malignant, 21 benign)	3 mg/kg	20 h
Holt et al., PLOS One 2014 [16]	8 dogs	Primary pulmonary tumors: adenocarcinoma (6), neuroendocrine tumor (1), squamous cell carcinoma (1)	5 mg/kg	24 h
Sakurai et al., Front Vet Sci 2023 [21]	40 dogs	Pulmonary tumors/nodules (44): adenocarcinoma (32), histiocytic sarcoma (5), undifferentiated sarcoma (3), epithelial metastases (2), carcinosarcoma (1), non-neoplastic (1)	2 mg/kg	12–24 h
Iida et al., J Small Anim Pract 2013 [23]	6 dogs	Hepatic masses/nodules (12): hepatocellular carcinoma (6), nodular hyperplasia (6)	0.5 mg/kg	12–18 h
Sakurai et al., BMC Vet Res 2022 [24]	104 dogs	Hepatic masses/nodules (122): hepatocellular carcinoma (76), hepatocellular adenoma (16), hyperplasia (12), sarcoma (5), hepatocholangiocarcinoma (4), abscess (2), bile duct adenoma (2), metastatic neoplasia (2), choledochal cyst (1), cholangiocarcinoma (1), neuroendocrine tumor (1)	0.5 mg/kg	12–24 h
Kim et al., Acta Vet 2025 [27]	1 dog	Intestinal adenocarcinoma	5 mg/kg	24 h

In summary, these published small animal surgical oncology studies demonstrate that, following preoperative IV ICG administration, delayed intraoperative NIR imaging can be utilized to identify primary tumors, but with variable performance depending on tumor type, location, and study design. Of note, no substantial adverse events related to IV ICG administration have been reported in any of these manuscripts. Findings of interest include that NIR fluorescence can extend beyond gross or microscopic tumor margins, suspected in association with optical diffusion, and there is potential for both false-positive and false-negative findings when assessing gross disease, surgical margins, and wound beds. In most studies, sensitivity for tumor detection has exceeded specificity for malignancy, with inflammatory tissues and benign lesions exhibiting fluorescence, though one hepatic study demonstrated comparatively higher specificity than sensitivity [23]. There appears to be limited reliability of ICG-based NIR imaging in determining histologic completeness of resection for pulmonary tumors, whereas performance appears more favorable for hepatic margin assessment (though ability of ICG to differentiate hepatic tumor type appears limited) [21,24]. Fluorescent lymph nodes have been inconsistently associated with metastatic disease, though relatively few studies have evaluated nodal NIR fluorescence and metastasis following IV administration of ICG. Ultimately, interpretation of findings in these studies is limited by small sample sizes, heterogeneous tumor populations, wide variations in ICG dosing and timing, inconsistent reporting of SBR, lack of standardized imaging protocols (with lack of reporting of select features in multiple studies), and potential for technical variability across imaging platforms. Notable knowledge gaps to date involve the absence of feline studies and limited data on lymph node and staging-directed applications.

#### 3.1.2. Selective NIR Agents

Due to the aforementioned limitations with IV ICG-based NIR imaging in surgical oncologic applications, there has been growing interest in the development of targeted and activatable NIR fluorescent probes. Multiple features that are unique to or upregulated by neoplastic tissue have been investigated as targets, such as folate receptors, integrins, endothelial and vascular growth factors, and proteases [1,2]. With these selective agents, fluorescent NIR molecules are conjugated to substrates that are overexpressed by neoplastic tissues, with the goal of enhanced and specific uptake in cancerous tissues as compared with non-neoplastic tissues [1,2]. Several agents are now FDA-approved, including OTL38 (a probe that targets folate receptors) for lung and ovarian cancer and pegulicianine (a cathepsin-activatable probe) for breast cancer [12]. Use of these agents in veterinary patients is still in the early stages of investigation, though several targeted NIR studies have identified the translational value of dogs as spontaneous large animal models of neoplasia, and these applications are likely to expand in an effort to benefit both companion animals and their human counterparts [28,29]. An updated review of the literature on targeted and activatable NIR fluorescent probes in small animal surgical oncology patients is summarized below and in Table 2.

A folate receptor-targeted NIR probe (OTL0038 [On Target Laboratories, LLC, West Lafayette, IN, USA]) has been assessed in 10 dogs with primary lung tumors [30]. In this study, all tumors fluoresced greater than the surrounding tissues (SBR 5.2 with Fluoptics system [Grenoble, France] and 1.9 with Storz system [Tuttlingen, Germany]) and NIR fluorescent margins were consistent with histologic margins [30]. In addition, in 3/10 dogs, NIR imaging identified fluorescent regional lymph nodes (SBR 2-5); these 3 nodes were extirpated and confirmed to be metastatic on histologic assessment [30]. In only one other dog, a non-fluorescent lymph node was identified and removed, and this node was not metastatic on histologic assessment [30]. Upon imaging the surgical site following pulmonary tumor resection, in 1/10 dog, fluorescence of the residual bronchus was identified; this tissue was resected and confirmed to be residual carcinoma [30].

Integrin-targeted NIR probes have been evaluated in several small animal surgical oncologic studies. One study on 24 dogs with a variety of superficial solid tumor types and locations utilized DA364 (Bracco Imaging SpA, Milan, Italy; an α_v_β_3_ integrin-specific probe) administered IV at various doses and intervals preoperatively [31]. In this study, a dose of 1.8 mg/m^2^ was identified as optimal for tumor delineation [31]. Although all tumors aside from one melanoma had an SBR of at least 1.0, visually distinct NIR fluorescence of the mass relative to adjacent tissues was evident in only 15/30 tumors, and no significant difference in SBRs was identified between malignant and benign masses [31]. When wound beds were evaluated for NIR fluorescence relative to histologic margins, true positives (NIR fluorescence with residual disease) were identified in 4 cases, false positives (NIR fluorescence without residual disease) were identified in 11 cases, true negatives (lack of NIR fluorescence without residual disease) were identified in 15 cases, and false negatives (lack of NIR fluorescence with residual disease) were not identified [31]. With regard to lymph nodes, the SBR was greater in metastatic nodes (3.28 ± 0.37) compared to non-metastatic nodes (1.58 ± 0.12) [31]. In a subsequent blinded study, 20 dogs with soft tissue sarcomas received either Angiostamp800 (Fluoptics; another anti-α_v_β_3_ integrin probe) (NIR group) or saline (control group) at a variety of intervals preoperatively [32]. All 10 tumors in the NIR group exhibited fluorescence with an SBR of 7.4 ± 5.8 in native biopsies (prior to formalin fixation), and the NIR signal correlated with histologic disease [32]. Moreover, resections were revised in 5/10 cases in the NIR group based on NIR findings, which resulted in complete resections that otherwise would have had incomplete margins in 4 of these dogs; in one of these cases, 2 fluorescent lymph nodes were identified and resected, both of which were confirmed to be metastatic on histopathology (one sarcoma and one carcinoma) [32]. Resections were incomplete in 1/10 dog in the NIR group and 2/10 dogs in the control group [32]. Finally, one study has evaluated the use of an anti-α_v_β_3_ integrin-targeted probe (AngioStamp [Fluoptics]) in 12 cats with infiltrative fibrosarcomas at a variety of doses and injection intervals [33]. Tumor fluorescence was identified in all cases, with the greatest SBR (14 ± 2.6) obtained at a dose of 0.3 mg/kg 36 h preoperatively [33]. No cases had residual positive NIR signal in the wound bed, and, of 11 cats that survived perioperatively, 1 cat died due to local recurrence and metastases 6 months postoperatively [33].

Finally, cathepsin-targeted NIR probes have been evaluated in several canine tumor applications. In an initial translational study, 4 dogs with primary pulmonary tumors underwent surgery for tumor resection after IV injection of VGT-309 (LI-COR Biosciences, Lincoln, NE, USA; UI Pharmaceuticals, Iowa City, IA, USA), a quenched activity-based, cathepsin-targeted NIR probe [28]. In this study, all tumors demonstrated enhanced fluorescence with in vivo SBRs ranging from 2.15 to 3.71 (with no significant difference between dose groups) and a correlation between NIR fluorescent and histologic margins [28]. Another study evaluated this same imaging agent (VGT-309 [Chinese Peptide Company, Hangzhou, Zhejiang, China; UI Pharmaceuticals) in 12 dogs with appendicular osteosarcoma undergoing limb amputation [34]. All bone tumors fluoresced on cross-section of the bone with mean SBR 3.55, and the extent of NIR fluorescence correlated with MRI (mean discrepancy of 6.5 mm) and histologic margins, though regions of necrotic tumor did not fluoresce; fluorescence was visible through cortical bone after soft tissue removal in all cases, but this consistently underestimated the intramedullary tumor extent [34]. Regional lymph nodes were evaluated in 6 cases and all were positive for fluorescence but had no histologic evidence of metastasis [34]. One final study evaluated the same imaging agent (VGT-309 [Vergent Bioscience, Minneapolis, MN, USA]) in 6 dogs with insulinoma [29]. Pancreatic insulinoma lesions had significantly greater mean fluorescence intensities than the surrounding pancreas with a median SBR of 1.906, and one dog had a small, occult pancreatic mass that was identified via NIR guidance but not via initial visual inspection or palpation of the pancreas; histologic tumor margins correlated with margins of NIR fluorescence [29]. Background fluorescence of liver and lymph nodes was observed in all cases, though only 1/6 dog was diagnosed with nodal and hepatic metastasis [29].

Overall, targeted and activatable NIR imaging probes have demonstrated promise with potential for intraoperative neoplastic disease identification in small animal cancer patients. Folate receptor-targeted and integrin (α_v_β_3_)-targeted probes have consistently resulted in tumor fluorescence across multiple tumor types, with several studies demonstrating close correlation between NIR fluorescence and histologic tumor margins, identification of occult residual disease, and detection of metastatic regional lymph nodes. In particular, targeted probes have altered surgical decision-making and improved margin completeness in soft tissue sarcoma and pulmonary tumor models [30,32]. Cathepsin-activated probes have also demonstrated the ability to delineate viable tumor tissue and correlate with histologic and advanced imaging margins, though background fluorescence of liver and lymph nodes and lack of fluorescence in necrotic tumor regions have been documented limitations. Across studies, no adverse events related to NIR agent administration have been reported. However, interpretation of results of these studies is limited by heterogeneity in imaging agents, dosing strategies and timing of administration, tumor types, NIR imaging systems and protocols, and methods used to confirm probe specificity and targeting. Background fluorescence and inconsistent performance for detection of metastatic disease, particularly within lymph nodes, remain important challenges. Despite these limitations, these early translational studies support the feasibility and safety of targeted and activatable NIR probes while underscoring the need for standardized protocols and larger, tumor-specific investigations in small animal oncology patients.

### 3.2. NIR Applications of Sentinel Lymph Node Mapping

An alternative application of intraoperative NIR imaging in both human and companion animal cancer patients involves SLN mapping. Multiple preoperative and intraoperative techniques for SLN mapping have been described. When intraoperative NIR imaging is used for SLN mapping, an NIR fluorescent probe, most commonly ICG, is applied locally immediately adjacent to or directly into a tumor, where it is then taken up by lymphatic vessels to the first tumor-draining lymph node, which is considered to be the SLN. This lymph node is identified in real time, often within minutes after ICG injection, as the first fluorescent lymph node with an NIR camera system and is generally selectively sampled or removed for staging aimed at detecting early nodal metastasis.

Identification of metastatic lymph nodes is important as a prognostic factor for many tumor types, and extirpation of metastatic nodes has the potential to improve outcomes in tumors such as canine apocrine gland anal sac adenocarcinoma (AGASACA) and mast cell tumors [35]. Importantly, it is well-documented that the lymphatic drainage pattern of tumors can vary substantially and that sampling the locoregional lymph node presumed to be at greatest risk for early metastatic disease is often inaccurate and not representative of the SLN in dogs and cats [35]. As compared to radical regional lymphadenectomy, SLN mapping is less invasive and reduces surgical morbidity and, potentially, anesthesia times [35]. Importantly, SLN mapping is primarily applicable in settings of potential occult metastatic nodal disease rather than effaced metastatic lymph nodes, as infiltration of neoplastic cells within a lymph node can result in obstruction of lymphatic flow and development of altered lymphatic routes, with subsequent false-negative SLN results [36,37,38].

Two preliminary studies in healthy dogs as a model for dogs with neoplasia evaluated SLN mapping via ICG administered transdermally (in the inguinal, axillary, and popliteal regions) and into the maxillary oral mucosa [39,40]. In these dogs, transcutaneous NIR imaging identified SLNs in 17/18 events following transdermal injection and 6/6 events following transmucosal injection [39,40]. These studies have prompted further evaluation of ICG-based SLN mapping in small animal cancer patients. An updated review of the literature on intraoperative SLN mapping using ICG-based NIR imaging in small animal surgical oncology patients is summarized below and in Table 3.

To date, in dogs, mast cell tumors represent the most commonly described tumor type with research and data on SLN mapping applications, largely owing to the frequency of this disease, the important role of nodal metastatic disease on prognosis in these dogs, and the documented therapeutic effect of extirpating metastatic nodes (particularly HN3 stage) in dogs with mast cell tumors [41,42]. In one study, 20 dogs with integumentary mast cell tumors (dermal, subcutaneous, and mucocutaneous) underwent preoperative SLN mapping via indirect CT lymphography (CTL) using iodinated contrast followed by intraoperative SLN mapping via NIR imaging [43]. SLN mapping via NIR imaging detected SLNs in 16/20 (80%) dogs at a median time of 60 s (range 10–360) [43]. Of the 4 dogs in which NIR-based SLN mapping failed, all had subcutaneous tumors [43]. When CTL and NIR imaging were compared, there was complete agreement in 8/20, partial agreement in 8/20, and no agreement in 1/20 [43]. When NIR-based SLN mapping was considered relative to the anatomic draining lymph node, there was complete agreement in 6/20, partial agreement in 9/20, and no agreement in 1/20 cases [43]. Importantly, 19/20 identified SLNs via NIR imaging had HN1-3 changes on histology [43]. In another recent manuscript comparing lymphoscintigraphy to NIR imaging for SLN mapping in 48 dogs with 60 mast cell tumors (cutaneous and subcutaneous), SLN mapping via NIR imaging identified an SLN in all 30 cases that underwent NIR imaging, and NIR imaging and lymphoscintigraphy showed total agreement in 23/30 cases and partial agreement in 7/30 cases, in which NIR imaging identified additional SLNs [44]. In a control group of 30 cases that underwent unguided lymphadenectomy (i.e., without intraoperative SLN mapping via NIR imaging), failure to identify a lymph node occurred in 4 cases, but rescue NIR imaging allowed for identification of the SLN and nodal extirpation in all these cases [44]. Sensitivity of NIR-based SLN mapping was 93.7% and negative predictive value ranged from 91.1% to 98.6% [44]. Moreover, surgical time and incision length were significantly shorter in the intraoperative NIR group compared to the control unguided lymphadenectomy group [44]. Finally, one additional study compared findings of NIR-based SLN mapping for guided nodal dissection to locoregional lymphadenectomy without SLN mapping in dogs with mast cell tumors [45]. In the NIR group (35 dogs), 70 locoregional lymph nodes were planned for dissection, and 57 of those were fluorescent; in 15/35 dogs, fluorescent nodes did not reflect the predicted locoregional node [45]. Nodal metastasis was identified in 53.4% (31/58) nodes dissected via NIR-based SLN mapping and only 32.0% (16/50) nodes dissected using locoregional dissection alone; metastatic disease was identified in 24/35 (68.6%) dogs that underwent SLN mapping via NIR imaging and 14/43 (32.6%) dogs that underwent locoregional lymphadenectomy alone [45].

Another described use of intraoperative SLN mapping with ICG and NIR imaging involves dogs with oral tumors, in which nodal staging is important for a variety of tumor types [38]. In one study, preoperative CTL, intraoperative ICG-based NIR imaging, and intraoperative methylene blue (visible dye) were compared as SLN mapping techniques [38]. Importantly, ipsilateral and contralateral lymphatic tracts and SLNs were identified in these dogs, and SLNs were predominantly localized to mandibular and medial retropharyngeal lymphocentrums [38]. Use of ICG resulted in a greater proportion of SLNs identified (52/57) compared with methylene blue (29/57) and CTL (24/57), and the level of agreement between the modalities was fair [38]. It was postulated that this result may have been caused by the rapid transit time of ICG with relatively quick uptake by SLNs as well as higher-tier lymph nodes [38]. However, only 1/57 SLN identified by any modality was metastatic on histologic assessment, and this node was not determined to be an SLN via ICG with NIR imaging [38]. It was hypothesized that the nodal metastasis may have altered lymphatic pressure and impacted ICG greater than iodinated contrast [38]. Based on this study, the authors recommended a combination of SLN mapping techniques to optimize SLN detection [38].

In a recent report evaluating preoperative lymphoscintigraphy and intraoperative ICG-based NIR imaging for SLN mapping in 50 dogs and 4 cats with a variety of tumor types (including scars without gross disease in multiple cases), the SLN detection rate with NIR imaging was 93%, which was similar to that of lymphoscintigraphy (92%) [46]. The only significant difference identified between techniques was a greater number of SLNs identified via NIR imaging, though the number of detected nodes with metastatic disease was similar [46]. Residual fluorescence detected by leakage of ICG after rupture of lymphatic vessels during dissection was recorded in 24.2% of cases [46]. Importantly, another study evaluated the use of SLN mapping with NIR imaging or other techniques in dogs with surgical scars after primary tumor excision [47]. In all cases in which ICG was used, SLNs were identified [47]. The location of the SLN did not correspond to the anatomical regional node for the majority of scars [47]. Additionally, in 8 of the NIR-based SLN mapping cases, the SLN was determined to be metastatic (all originally had mast cell tumors) [47]. Though none of these dogs had SLN mapping performed prior to the initial mass excision to assess for consistency between SLN mapping results in the setting of gross disease as compared to the postoperative scar, the authors concluded that SLN mapping of a surgical scar is feasible and should be considered (especially in mast cell tumor cases) given the potential for identification of metastatic SLNs [47]. However, other studies (utilizing other SLN mapping techniques without NIR imaging) have demonstrated that surgery with mass removal can affect the lymphatic patterns and results of SLN mapping postoperatively, such that this is important to consider in the setting of SLN mapping for surgical scars following tumor removal [48,49].

A recent study evaluated the use of intraoperative SLN mapping via ICG (with a variety of concentrations used) in dogs with mammary tumors [50]. Fluorescence was visible within lymphatics within 5 min of injection, and the detection rate of SLNs was 94.4% (34/36 lymph nodes) [50]. An ICG concentration of 2.5 mg/mL resulted in the greatest mean lymphatic fluorescence, and a concentration of 1.0 mg/mL resulted in the greatest mean lymph node fluorescence and the shortest surgical times [50]. Importantly, contralateral lymphatic drainage occurred in 4/36 cases, and tumors in the third mammary gland exhibited highly variable drainage patterns [50].

Canine AGASACA represents another described application of SLN mapping with ICG [51]. For dogs with AGASACA, nodal metastasis occurs with relatively high frequency and impacts prognosis, and extirpation of metastatic nodes has been associated with improved outcomes [51]. The iliosacral lymphocentrum is the most common location for initial metastatic disease in these dogs, though drainage from the primary anal sac tumor to specific lymph nodes within this center is variable [52]. In a study on 8 dogs with unilateral AGASACA and no overt metastatic disease on initial staging, a variety of SLN mapping modalities were compared: preoperative contrast-enhanced ultrasound (CEUS) with transrectal ultrasound, preoperative CTL, and laparotomy with intraoperative methylene blue and ICG-based NIR imaging [51]. In all dogs, CTL and intraoperative SLN mapping with ICG and methylene blue identified at least one SLN, though pre- and intra-operative findings partially differed in 4/8 dogs; while preoperative CEUS identified an SLN in 7/8 dogs, the exact SLN location was not able to be determined with this technique alone [51]. In 2/8 dogs, extirpated iliosacral lymph nodes were identified to be metastatic, and all 7 metastatic nodes were SLNs; 6/7 metastatic nodes were identified as sentinel on both CTL and intraoperative SLN mapping (with both ICG and methylene blue), though 1/7 metastatic node was identified as sentinel only via intraoperative mapping (with both ICG and methylene blue) [51]. The authors concluded that a combination of preoperative CTL and intraoperative mapping with ICG and methylene blue was beneficial due to unique advantages of each modality, potential for divergent findings between modalities, and the resulting identification and safe extirpation of metastatic nodes in dogs with occult metastatic AGASACA [51]. Intraoperative SLN mapping was valuable in identifying SLNs and guiding dissection for safe and efficient extirpation of small nodes deep to retroperitoneal fat and adjacent to important vascular structures, with ICG and NIR imaging quickly identifying lymphatic tracts and fluorescence in the region of the SLNs [51].

Though no major complications with SLN mapping occurred, the authors noted that identification of minimally invasive techniques that allow access to the entire iliosacral lymphocentrum for SLN mapping (given the potential for variable and both ipsilateral and contralateral lymphatic drainage) would be beneficial in reducing surgical morbidity in dogs with AGASACA [51]. A recent study described a minimally invasive approach via lateral recumbency with ICG/NIR-guided iliosacral lymph node extirpation in dogs with a variety of tumor types (including two with AGASACA); however, only ipsilateral and predominantly medial iliac lymph nodes were extirpated, and the authors noted that the use of ICG and NIR imaging in these cases was intended to guide targeted nodal resection minimally invasively, but the technique used was not truly representative of SLN mapping given the inability to evaluate the entire iliosacral lymphocentrum with their approach [53]. A recent canine cadaveric study identified a novel minimally invasive approach via supported sternal recumbency to be effective in identifying and extirpating SLNs throughout the iliosacral lymphocentrum (with preoperative CTL and intraoperative ICG-based NIR imaging used for SLN mapping of the grossly normal anal sac) [54]. A clinical trial is ongoing at the author’s institution to assess MIS via supported sternal recumbency for intraoperative ICG/NIR-guided SLN mapping and extirpation in dogs with AGASACA.

Another described application of intraoperative SLN mapping using ICG and NIR imaging in small animal surgical oncology is in canine primary pulmonary neoplasia. In dogs with primary pulmonary tumors, nodal staging is important due to the high incidence and prognostic significance of nodal metastasis [55]. A case report described an intercostal, MIS approach (video-assisted thoracoscopic surgery) for SLN mapping with intraparenchymal, peritumoral injection of ICG in a dog with a right caudal pulmonary mass (adenocarcinoma) with no overt metastatic disease on preoperative CT [55]. In this dog, NIR imaging identified the right tracheobronchial node to be sentinel, and this node was extirpated and determined to be reactive on histology [55]. In a recent abstract, 7 dogs that underwent preoperative CTL followed by intraoperative SLN mapping with ICG-based NIR imaging for primary pulmonary tumors were described [56]. It was reported that CTL identified an SLN in 2/7 dogs, and ICG-based NIR imaging identified an SLN in all dogs (which included the 2 SLNs identified on CTL); histology identified metastatic nodal disease in 2/7 cases [56]. Locations of the SLN included tracheobronchial nodes in 5 dogs, a sternal node in 1 dog, and paraesophageal or retrotracheal node in 1 dog, and the SLN was ipsilateral to the tumor or centrally located in all dogs; however, the contralateral thorax was not inspected intraoperatively [56]. Leakage of ICG was noted in 3 dogs intraoperatively [56]. In another recent abstract, 21 healthy research dogs (as a model for dogs with pulmonary tumors) underwent intercostal thoracoscopy with ICG injection into the ipsilateral caudal lung lobe; dogs were randomized to receive one-lung ventilation or routine ventilation [57]. An SLN was identified in 19/21 dogs (including 8/12 dogs that received one-lung ventilation and 9/9 dogs that were ventilated routinely), and time to detection of the SLN was delayed in cases undergoing one-lung ventilation [57]. In 11 dogs, the SLN included a tracheobronchial node, while in 8 dogs, at least one SLN was identified as a cranial mediastinal or sternal node, highlighting the potential variability in lymphatic drainage from normal canine pulmonary tissues [57]. Importantly, none of these studies have evaluated the contralateral hemithorax intraoperatively, such that potential for contralateral lymphatic drainage and ICG nodal uptake may have occurred but went undetected. However, the potential for contralateral nodal drainage and metastasis in dogs with primary lung tumors is not well-described to date.

The use of ICG for SLN mapping in dogs with thyroid tumors without evidence of metastasis on routine staging has also been described [58]. The potential for anatomical variation in locoregional draining lymph nodes, lack of sensitivity of routine imaging modalities in identifying early nodal metastasis, potential for high incidence of nodal metastasis with routine nodal staging, and currently unknown significance of nodal metastasis in dogs with thyroid carcinoma all point to a need for development of standardized techniques for nodal staging in this disease [58]. In 6 dogs that underwent preoperative CTL and intraoperative SLN mapping with methylene blue and ICG with NIR imaging, at least one SLN was identified with both pre- and intra-operative mapping, and these results completely agreed in 3/6 cases, partially agreed in 2/6 cases, and diverged in 1/6 case [58]. Identified SLNs included medial retropharyngeal, cranial deep cervical, and superficial cervical nodes, and SLNs were bilateral in 1/6 dog and ipsilateral to the tumor in 5/6 dogs [58]. In 2/6 dogs, extirpated SLNs (3 total nodes) were metastatic on histologic evaluation; these included 1 node that was identified to be sentinel on pre- and intra-operative imaging and 2 nodes that were identified to be sentinel on intraoperative imaging only [58]. Based on these findings, the authors concluded that a combination of SLN mapping techniques was beneficial owing to the unique advantages and complementary nature of techniques [58]. Intraoperative mapping with ICG was useful in initial intraoperative identification of the SLN and guidance of dissection for safe and efficient extirpation of small nodes adjacent to important vascular structures; when the OR and room lights were required and NIR guidance was no longer feasible with the NIR imaging equipment utilized in this study, methylene blue became useful to guide dissection under visible light [58]. Importantly, the authors also described a benefit to intraoperative SLN injection protocol alterations–smaller injection volume and injection immediately after ventral cervical approach and prior to dissection resulted in reduced dispersion of ICG and methylene blue and improved the ability to identify lymphatic tracts and SLNs intraoperatively [58].

Historical research has evaluated intraoperative SLN mapping utilizing alternative (non-ICG based) NIR agents in dogs with bladder transitional cell carcinoma (urothelial carcinoma) [59]. In this study, following investigation in healthy dogs and pigs, 5 clinical dogs with transitional cell carcinoma underwent SLN mapping immediately prior to euthanasia [59]. Human serum albumin conjugated with HSA800 (LI-COR Biosciences) was utilized as the NIR imaging agent in clinical dogs of this study [59]. In one dog, the imaging agent was injected via cystourethroscopy around a urethral tumor, and, in 4 dogs, the imaging agent was injected via laparotomy into the bladder wall from the serosal surface [59]. The authors commented that intratumoral injection in cases not described resulted in ineffective SLN mapping with no migration into lymphatics, but peritumoral injection resulted in fluorescence of lymphatics (5/5) and SLNs (4/5) [59]. In 4/5 dogs, 1–2 fluorescent nodes were identified, and all 4 of these dogs had nodal metastasis on necropsy (only involving the fluorescent nodes in 1 dog and involving the fluorescent nodes as well as non-fluorescent nodes in 3 dogs) [59]. To date, ICG has not been reported for intraoperative NIR-based SLN mapping in dogs with bladder tumors undergoing surgery for treatment or staging of their disease.

A single case report has been published on intraoperative SLN mapping with ICG and NIR imaging in a dog with a primary insulinoma in the left limb of the pancreas [60]. In this dog, intratumoral injection of ICG resulted in rapid lymphatic fluorescence and the identification of 6 SLNs, including jejunal, right colic, duodenal, right and left hepatic, and splenic nodes; 3 of these nodes were not clearly identified prior to NIR imaging [60]. These nodes were all extirpated, and none had evidence of metastatic disease [60]. In addition, the same case report that described preoperative systemic ICG administration in a dog with intestinal adenocarcinoma also described the use of local ICG administration for SLN mapping [27]. In this dog, lymphatic drainage and SLN identification via NIR imaging occurred within 2 min of peritumoral injection, and a single, enlarged lymph node (16 mm × 15 mm) exhibited fluorescence [27]. This lymph node as well as one other lymph node identified to be enlarged on CT (but smaller than the fluorescent node) were extirpated, and only the larger, fluorescent node contained metastatic disease (though the non-metastatic node was composed of well-differentiated adipose tissue on histologic assessment) [27].

Finally, to date, several manuscripts have been published documenting intraoperative SLN mapping via ICG with NIR imaging in cats. In one study, a simulated feline tumor model was utilized to evaluate the effect of volume and methylene blue on fluorescence intensity and transit of ICG for SLN mapping [61]. In 6 healthy cats that were randomly divided into two groups (ICG 2.5 mg/mL alone or ICG in combination with methylene blue 10 mg/mL), 1 or 2 mL solutions were injected intradermally in 4 quadrants around a simulated tumor on both pelvic limbs of all cats; one additional cat served as a control with a single pelvic limb injected with ICG alone and the other pelvic limb injected with methylene blue alone [61]. In these cats, a larger solution volume, but not solution content (ICG alone vs. ICG with methylene blue), was associated with decreased transit times to the SLN [61]. Fluorescence intensity of the SLN was greater when ICG was used alone as compared to ICG in combination with methylene blue, but the solution volume did not influence fluorescence intensity [60]. No adverse events were documented in these cats [61]. Thus far, ICG-based SLN mapping has only been reported in two publications on clinical cats with neoplasia [62]. In one study, 14 cats with a variety of tumor types were included, 9 of which underwent intraoperative SLN mapping via ICG and NIR imaging and 5 of which underwent lymphoscintigraphy and methylene blue injection for SLN mapping [62]. SLNs were identified in all cases, and a total of 14 SLNs were identified in the 9 cats that underwent NIR imaging [62]. Nodal metastasis was histologically detected in 8/25 SLNs extirpated from 5/12 cats, all of which had mast cell tumors [62]. Of all 14 cases, the SLN did not completely correspond to the locoregional lymph node in 71.4%. In one other case report, intraoperative SLN mapping with ICG was described in a cat with three cutaneous, low-grade mast cell tumors [63]. ICG-based SLN mapping was successful in identifying the SLN(s) for each tumor and guiding extirpation [63]. Four nodes were fluorescent, and 5 nodes were extirpated; all fluorescent nodes were consistent with canine Weishaar grades of HN1 or HN2 stage (though this staging scheme has not been validated for cats), and the non-fluorescent node was non-metastatic [63]. No adverse events were documented in any cats of these studies [62,63].

**Table 3:** Small animal surgical oncology publications evaluating intraoperative SLN mapping using NIR imaging.

Manuscript	Species and Number of Animals	Tumor Type	SLN Mapping Techniques Employed	Protocol Used for SLN Mapping via NIR Imaging
Alvarez-Sanchez et al., Vet Surg 2023 [43]	20 dogs	Mast cell tumors: dermal (10), subcutaneous (8), mucocutaneous (2)	Preoperative CTL, intraoperative NIR	1 mL ICG (2.5 mg/mL) mixed with 4 mL dextrose 5% in water (0.5 mg/mL ICG): administered 1 mL peritumorally in 4 quadrant technique; extirpated all SLNs identified via CTL and NIR as well as the anatomic draining lymph node
Chiti et al., Vet Comp Oncol 2025 [44]	48 dogs	Mast cell tumors (60): cutaneous (45), subcutaneous (15)	Preoperative lymphoscintigraphy in all dogs; treatment group (30 dogs): intraoperative NIR; control group (30 dogs): no intraoperative SLN mapping, unguided regional lymph node extirpation	ICG (2.5 mg/mL) 0.25–1 mL injection volume total (median 0.45 mL) in 4 quadrants peritumorally (intradermally for dermal tumors, subcutaneously for subcutaneous tumors)
Beer et al., J Small Anim Pract 2022 [45]	35 dogs	Mast cell tumors (60 primary tumors, 18 scars)	Lymphadenectomy performed with NIR SLN mapping (35 dogs) or standard regional lymphadenectomy (43 dogs)	ICG (5 mg/mL) 0.5–1 mL total volume administered in 4 quadrants peritumorally into subcutaneous/dermal tissue immediately adjacent to tumor or scar
Wan et al., Vet Comp Oncol 2021 [38]	14 dogs	Oral tumors: osteosarcoma (4), ameloblastoma (2), fibrosarcoma (1), squamous cell carcinoma (1), melanoma (1), carcinoma of unknown origin (1), periodontal sarcoma (1), spindle cell sarcoma (1), ameloblastic fibroma (1), gingival fibrous hyperplasia (1)	Preoperative CTL, intraoperative NIR and methylene blue SLN mapping	Solution of 0.1 mL (2.5 mg/mL) ICG + 0.1 mL methylene blue (10 mg/mL) + 0.8 mL saline injected peritumorally submucosally in 3–4 equal parts around the tumor
Gariboldi et al., BMC Vet Res 2025 [46]	50 dogs, 4 cats (60 tumors total)	Variety of superficial solid tumor types (dogs: 26 cutaneous mast cell tumors, 6 subcutaneous mast cell tumors, 9 mammary carcinomas, 2 soft tissue sarcomas, 1 oral fibrosarcoma, also scars in 12 [7 cutaneous mast cell tumors, 2 subcutaneous mast cell tumors, 2 oral melanomas, 1 soft tissue sarcoma]; cats: 2 cutaneous mast cell tumors, also scars in 2 [cutaneous mast cell tumor in 1, mammary carcinoma in 1])	Preoperative planar lymphoscintigraphy and intraoperative NIR SLN mapping	ICG 0.2 mL (2.5 mg/mL) + 0.2 mL saline (total volume 0.4 mL) injected in 4 quadrants peritumorally or around the scar
Gariboldi et al., Animals 2022 [47]	33 dogs (34 scars)	Variety of initial primary tumor types: mast cell tumor (29), soft tissue sarcoma (2), oral melanoma (1), subungual melanoma (1), mammary adenocarcinoma (1)	SLN mapping guided by radiopharmaceutical and methylene blue in 22/34 cases, NIR imaging in 10/34 cases, and a combination of both techniques in 2/34 cases	ICG (concentration and volume not provided) injected in 2–4 quadrants around the scar
Kim and Lee, Sci Rep 2025 [50]	24 dogs	Mammary tumors (1–3 per dog; carcinoma in 12, osteosarcoma in 1, carcinosarcoma or spindle cell carcinoma in 2, and only benign tumors in 9)	Intraoperative NIR SLN mapping	Randomly assigned ICG concentration groups (0.5, 1.0, and 2.5 mg/mL), total volume 0.5 mL of given concentration injected peritumorally in 4 quadrants
Griffin et al., Vet Oncol 2024 [51]	8 dogs	Unilateral AGASACA	Preoperative CEUS with transrectal ultrasound, preoperative CTL, and intraoperative SLN mapping via methylene blue and ICG-based NIR imaging	ICG 0.2 mL (2.5 mg/mL) combined with methylene blue 0.4 mL (5.0 mg/mL) and 0.4 mL sterile saline, injected in 4 quadrants peritumorally
Griffin et al., Vet Surg 2025 [55]	1 dog	Pulmonary adenocarcinoma	Intraoperative ICG-based NIR imaging	ICG 1 mL (2.5 mg/mL) administered percutaneously under thoracoscopic-guidance into the lung tissue immediately adjacent to the gross mass via a single injection
Griffin et al., Vet Surg 2025 [58]	6 dogs	Unilateral thyroid carcinoma	Preoperative CTL, SLN mapping via methylene blue and ICG-based NIR imaging	Dogs 1–2: 1 mL solution of 0.2 mL ICG (2.5 mg/mL) + 0.4 mL methylene blue (5 mg/mL) + 0.4 mL saline; injected percutaneous, peritumoral, 4 quadrant, 25 Ga needle Dog 3: same solution as dogs 1–2; injected peritumoral, 4 quadrant, 25 Ga needle, following ventral cervical approach and prior to dissection Dogs 4–6: 0.3 mL solution of 0.1 mL ICG (5 mg/mL) + 0.2 mL methylene blue (10 mg/mL); same injection timing as dog 3, but with 27 Ga needle
Knapp et al., Eur Urol 2007 [59]	5 dogs	Invasive transitional cell carcinoma	Intraoperative SLN mapping with non-ICG-based NIR imaging agent	NIR imaging agent HSA800 (human serum albumin conjugated with IRDye 800CW): 0.2–0.3 mL per injection site administered peritumorally in 1 or multiple injection sites, trans-serosally in 4 dogs (during laparotomy) and trans-mucosally in 1 dog (during cystourethroscopy)
Nolff et al., Front Vet Sci 2023 [60]	1 dog	Insulinoma	Intraoperative ICG-based NIR imaging	0.2 mL ICG (2.5 mg/mL) injected intratumorally
Kim et al., Acta Vet 2025 [27]	1 dog	Intestinal adenocarcinoma	Intraoperative ICG-based NIR imaging (also preoperative IV ICG)	0.5 mL ICG (2 mg/mL) injected into the submucosal layer around the tumor
Chiti et al., Animals 2022 [62]	12 cats	14 solid tumors: mast cell tumors (9), oral squamous cell carcinoma (2), oral fibrosarcoma (1), salivary adenocarcinoma (1), and ceruminous adenocarcinoma (1)	Intraoperative lymphoscintigraphy and methylene blue in 5/14; intraoperative ICG-based NIR imaging in 9/14	ICG (volume and concentration not reported) injected peritumorally or intratumorally
Arz et al., J Feline Med Surg 2022 [63]	1 cat	3 cutaneous mast cell tumors	Intraoperative ICG-based NIR imaging	1.5 mL ICG (5 mg/mL) injected in 4 quadrants peritumorally around each tumor

In summary, NIR imaging, predominantly utilizing ICG as an imaging agent, for intraoperative SLN mapping appears to be safe and feasible in both dogs and cats and is increasingly recognized as a valuable adjunct for oncologic staging across multiple tumor types. The high SLN detection rate, greater tissue penetration compared to visible dyes such as methylene blue, and excellent safety profile have contributed to the growing use and applications of ICG-based NIR imaging for SLN mapping in companion animals [64]. However, despite encouraging findings, several limitations and unresolved questions remain. Across tumor types, SLN mapping, including with ICG and NIR imaging guidance, represents an important staging shift in that it enables earlier identification and removal of occult metastatic lymph nodes that have not previously been recognized or reported in staging algorithms and prognostic outcomes; thus, the prognostic and therapeutic impact of this approach remains incompletely defined for many diseases, including canine AGASACA, pulmonary, and thyroid tumors and feline mast cell tumors. Interpretation of the existing literature is further complicated by substantial heterogeneity in tumor types and locations included, overall SLN mapping techniques utilized or combined, NIR imaging protocols and equipment, ICG concentrations and volumes used, and injection location, thereby limiting direct comparison across studies. Technical challenges of NIR-based SLN mapping include restricted depth of fluorescence penetration and the surgical field of view, rapid lymphatic transit of ICG with potential identification of higher-tier rather than truly sentinel nodes, ICG leakage during dissection with contamination of the field, and potential impact of procedural factors such as ventilation strategy during thoracic mapping. Collectively, these findings underscore the need for standardized, tumor-specific SLN mapping protocols and prospective studies designed to clarify the prognostic and therapeutic implications of NIR-guided SLN extirpation in small animal oncology patients.

### 3.3. Other Applications of NIR Imaging in Small Animal Surgical Oncology

Although the predominant applications of NIR imaging in small animal oncology have focused on IV administration for intraoperative localization of primary and metastatic disease, as well as local administration for intraoperative SLN mapping, other applications have been described less frequently. One effort has been aimed at the intraoperative detection of normal parathyroid glands in dogs. A recent case series described two dogs that underwent bilateral thyroidectomy for thyroid neoplasia with parathyroid preservation [65]. In these dogs, IV ICG 0.02 mg/kg was administered twice following thyroid exposure with immediate NIR fluorescence of the external parathyroid gland [65]. However, fluorescence of both the parathyroid gland and thyroid tissue was evident in these cases, and the distinction between parathyroid and thyroid tissue based on differential fluorescence may not be feasible in all cases [65]. Furthermore, despite parathyroid preservation, both dogs developed mild hypocalcemia postoperatively, with one dog requiring long-term supplementation [65]. In addition, in one of these dogs, postoperative cervical CTs identified contrast-enhancing tissue in the region of the excised thyroid gland, representing possible residual thyroid tissue vs. recurrent disease [65]. Thus, when preserving parathyroid tissue in the setting of thyroid neoplasia, efforts should be made not to compromise margins of the thyroidectomy with the potential for residual disease. Prior to this case series, two other reports characterized IV ICG administration (at doses of 0.0125–0.4 mg/kg) for the NIR imaging of normal parathyroid tissues in research dogs [66,67]. In these dogs, background fluorescence intensity of the thyroid tissue was noted, such that ICG-based NIR imaging did not consistently distinguish normal parathyroid from thyroid glands [66,67]. Overall, further investigation is warranted to clarify the clinical utility of IV ICG-based NIR imaging for parathyroid identification in dogs, including its reliability for differentiating parathyroid from normal or neoplastic thyroid tissue, its impact on intraoperative decision-making, and the implications of parathyroid preservation in bilateral thyroidectomy cases on both oncologic margin adequacy and postoperative calcium homeostasis. One alternative potential parathyroid detection-based use of ICG/NIR imaging in dogs includes the identification of hyperfunctional parathyroid glands in dogs with primary hyperparathyroidism. In the author’s experience, IV administration of 0.5 mg/kg ICG intraoperatively has resulted in inconsistent fluorescence of the hyperfunctional gland relative to adjacent thyroid tissue, and further study is needed to determine the utility of ICG-based NIR imaging in these dogs [68].

Another recently described application of NIR imaging involves delineation of the biliary tract to confirm patency in a dog undergoing cholecystectomy for a gallbladder neck tumor (leiomyosarcoma) [69]. In this dog, ICG 0.05 mg/kg was injected IV 45 min preoperatively for intraoperative cholangiography via NIR imaging, due to the rapid excretion of ICG in the biliary system [69]. Fluorescence was observed within the gallbladder, with a filling defect at the site of the tumor, and common bile duct, confirming ductal patency prior to cholecystectomy and tumor excision [69]. This case highlights the feasibility of NIR cholangiography in dogs; however, further study is needed to determine its clinical utility and consistency for the assessment of biliary anatomy and patency in small animal oncologic surgery applications.

An additional recent case report described the intraoperative submucosal injection of ICG with NIR imaging to localize and delineate a gastric leiomyosarcoma in a dog with a tumor located near the esophagogastric junction [70]. Following gastrotomy at the cardia, ICG was injected submucosally around the tumor (total volume 1 mL of 0.5 mg/mL solution), and, after approximately 30 min, NIR fluorescence of the tumor was noted to aid in the localization of the tumor and its margins for marginal resection [70]. Although this case illustrates the technical feasibility of submucosal ICG injection with subsequent NIR visualization, its practical impact on surgical decision-making is uncertain, as tumor localization appeared achievable prior to ICG administration (with ICG administration performed peritumorally), and the submucosal delivery method may result in non-specific peritumoral fluorescence rather than tumor-selective uptake and fluorescence, which could limit accuracy in margin assessment.

## 4. Conclusions and Future Directions

In summary, NIR imaging has emerged as a valuable adjunct in small animal surgical oncology, with growing applications in neoplastic disease localization, margin assessment, and SLN mapping. As demonstrated in this review, the majority of current veterinary applications rely on ICG, a nonselective fluorophore with a favorable safety profile and broad applicability with both systemic and localized administration. However, important limitations persist, including the limited NIR tissue penetration depth, lack of tumor specificity with potential for false-positive fluorescence (such as with inflammation), and inconsistent performance across tumor types. Additionally, the absence of standardized imaging protocols—with variability in the ICG dose, timing of administration, and imaging platforms and settings utilized—limits reproducibility and comparisons between studies.

Experiences from human surgical oncology provide important insights for the continued development of NIR imaging in veterinary medicine. In particular, intraoperative NIR imaging has become increasingly integrated into MIS applications in human patients, in which the lack of tactile feedback and common reliance on two-dimensional visualization present inherent challenges to tumor localization and margin assessment [11,71]. These same challenges are directly translatable to veterinary MIS procedures, highlighting a critical opportunity to expand NIR-guided applications in this space. Beyond its use for the delineation of neoplastic tissues for resection, NIR imaging may also be used to facilitate the visualization of critical normal structures (such as ureters and nerves), which may be particularly valuable as more complex procedures are performed using MIS techniques.

Despite its current utility, ICG-based imaging represents only an early phase of fluorescence-guided surgery. Targeted and activatable NIR probes offer the potential to improve specificity by selectively accumulating in neoplastic tissues. In human precision surgery initiatives, these agents are being developed to address key intraoperative endpoints, including accurate disease localization and margin identification, detection of occult synchronous lesions, enhanced surgical cytoreduction, identification of metastatic lymph nodes, and preservation of critical normal anatomy [12]. Continued investigation is needed to determine how these advances impact clinical outcomes and how they can be adapted for veterinary patients and vice versa, as dogs and cats represent important large-animal translational models of spontaneous neoplastic disease.

In addition, SLN mapping represents a paradigm shift in oncologic staging in veterinary medicine, allowing for the identification and removal of occult metastatic nodes not reliably detected through conventional staging modalities. However, while SLN mapping is frequently successful in identifying sentinel nodes, current techniques—including NIR imaging with ICG—do not reliably differentiate metastatic from non-metastatic lymph nodes intraoperatively. Emerging approaches, including targeted SLN mapping agents designed to bind tumor-specific biomarkers, may help overcome these limitations and improve intraoperative decision-making [64]. In parallel, further investigation is needed to determine the therapeutic impact of SLN extirpation in a variety of tumor types, including canine AGASACA, melanoma, thyroid carcinoma, primary pulmonary tumors, and other malignancies.

Additional technological advancements may further expand the capabilities of fluorescence-guided oncologic surgery in small animal patients. Recent investigations of imaging in the second near-infrared window (NIR-II characterized by 900–1880 nm wavelengths) have shown improved tissue penetration, higher resolution, and reduced background fluorescence compared to conventional NIR imaging, though much research in this field is still needed in clinical settings [72]. In addition, nanoparticle-based contrast agents, such as dual-modality liposomal platforms co-encapsulating iodinated contrast and ICG, offer the potential for combined preoperative and intraoperative SLN and tumor imaging [73,74]. Early studies in healthy dogs have demonstrated feasibility and safety with the use of these nanoparticles, though a clinical assessment is still needed [73,74].

Despite the encouraging progress, significant gaps remain. The data in small animals, particularly cats, are limited, and most studies involve small, heterogeneous populations with variable methodologies. Standardization of protocols for both systemic and local NIR applications (including injection location, dose, dilution, timing, and intraoperative NIR imaging techniques) will be critical to advancing the field. Prospective, tumor-specific studies with long-term follow-up are needed to identify indications, optimize techniques, and determine the impact of NIR-guided interventions on surgical decision-making, morbidity, and long-term oncologic outcomes in small animal cancer patients.

Overall, NIR imaging represents a powerful and evolving tool in companion animal surgical oncology. While current applications demonstrate feasibility and clinical promise, the continued refinement of imaging agents, standardization of protocols, and expansion into targeted and precision-based approaches are needed to fully realize its potential to improve surgical accuracy and patient outcomes.

## Figures and Tables

**Table 2:** Small animal surgical oncology publications evaluating IV administration of targeted and activatable fluorescent probes with subsequent intraoperative NIR imaging.

Manuscript	Species and Number of Animals	Tumor Type	NIR Imaging Agent	Mechanism of Action	IV Dose of Agent and Duration Between Injection and Surgery
Keating et al., Cancer 2017 [30]	10 dogs	Primary pulmonary tumors (carcinomas and adenocarcinomas)	OTL0038	Folate receptor-targeted	0.185 mg/kg, 2–3 h
Favril et al., Oncotarget 2020 [31]	24 dogs	Mammary tumors (adenocarcinoma in 6, adenoma in 2, cysts in 1), mast cell tumors (9), vaginal leiomyosarcoma (1), soft tissue sarcoma (1), osteosarcoma (1), cutaneous melanoma (1), apocrine gland adenocarcinoma (1), lipoma (1); several dogs had additional concurrent tumors	DA364	Integrin (αvβ3)-targeted	Various doses (0.6 mg/m^2^, 1.8 mg/m^2^, or 3.0 mg/m^2^), 24 or 48 h
Beer et al., Eur J Nucl Med Mol Imaging 2025 [32]	20 dogs: 10 received placebo (control group), 10 received NIR agent (NIR group)	Soft tissue sarcomas	Angiostamp800	Integrin (αvβ3)-targeted	0.15 mg/kg (or 0.15 mL/kg saline), various intervals: 36 h preoperatively in 3 dogs (2 control, 1 NIR), 6.5 h preoperatively in 1 dog (NIR), 12 h preoperatively in remaining 16 dogs
Wenk et al., Cancer Lett 2013 [33]	12 cats	Infiltrative fibrosarcoma	AngioStamp	Integrin (αvβ3)-targeted	Various doses, various intervals: 0.6 mg/kg 16 h preoperative (1 cat), 0.3 mg/kg 16 h preoperatively (4 cats), 0.3 mg/kg 24 h preoperatively (2 cats), 0.3 mg/kg 36 h preoperatively (3 cats), and 0.15 mg/kg 36 h preoperatively (2 cats)
Kennedy, Clin Cancer Res 2022 [28]	4 dogs	Primary pulmonary tumor (carcinoma or adenocarcinoma)	VGT-309	Cathepsin-targeted (quenched activity-based probe)	0.1 mg/kg or 0.3 mg/kg, 3 h or 18 h
Massie et al., Clin Cancer Res 2026 [34]	12 dogs	Appendicular osteosarcoma	VGT-309	Cathepsin-targeted (quenched activity-based probe)	0.2 mg/kg, 16–20 h
Griffin et al., PLOS One 2026 [29]	6 dogs	Insulinoma	VGT-309	Cathepsin-targeted (quenched activity-based probe)	0.2 mg/kg, 18–24 h (5 dogs) or 48 h (1 dog)

## Data Availability

No new data were created or analyzed in this study. Data sharing is not applicable to this article.

## Abbreviations

The following abbreviations are used in this manuscript:

AGASACA	Apocrine gland anal sac adenocarcinoma
CEUS	Contrast-enhanced ultrasound
CTL	Computed tomography lymphography
EPR	Enhanced permeation and retention
ICG	Indocyanine green
MIS	Minimally invasive surgery
NIR	Near-infrared
SBR	Signal-to-background ratio
SLN	Sentinel lymph node
SWIG	Second window indocyanine green
